# 4-Diethyl­amino-3,5-diisopropyl­benzalde­hyde

**DOI:** 10.1107/S1600536811047672

**Published:** 2011-11-16

**Authors:** Christoph Wink, Dieter Schollmeyer, Heiner Detert

**Affiliations:** a University Mainz, Duesbergweg 10-14, 55099 Mainz, Germany

## Abstract

The title benzaldehyde, C_17_H_27_NO, was prepared *via* lithia­tion of bromoaniline and reaction with DMF. In the crystal, the molecule adopts a *C*2-symmetrical conformation; nevertheless, two modes of disorder are present: the orientation of the aldehyde group (occupancy ratio 0.5:0.5) and of symmetry-equivalent ethyl groups [occupancy ratio 0.595 (7):0.405 (7)]. The phenyl­ene ring and the carbonyl group are essentially coplanar [C—C—C—O torsion angle = −179.0 (4)°] but the dihedral angle between the mean planes of the phenyl­ene ring and the amino group = 67.5 (2)°. This and the long [1.414 (3) Å] aniline C—N bond indicate electronic decoupling between the carbonyl and amino groups. The angle sum of 359.9 (2)° around the N atom results from steric compression-induced rehybridization.

## Related literature

The title compound was prepared as an inter­mediate in the synthesis of highly solvatochromic (Detert *et al.*, 2002 ▶; Detert & Schmitt, 2006 ▶) or acidochromic fluoro­phores (Schmitt *et al.*, 2008 ▶, 2011 ▶). For crystal structures of anilines with a *p*-accetor substituent, see: Fischer *et al.* (2011 ▶); Moschel *et al.* (2011 ▶). Acceptor-substituted anilines display dual fluorescence due to the formation of TICT (twisted intra­molecular charge-transfer) states, see: Rotkiewicz *et al.* (1973 ▶); Okada *et al.* (1999 ▶). For the crystal structure of 4-dimethyl­amino­benzaldehyde, see: Gao & Zhu (2008 ▶).
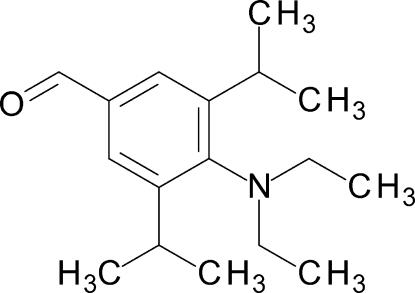


## Experimental

### 

#### Crystal data

C_17_H_27_NO
*M* *_r_* = 261.40
Monoclinic, 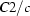

*a* = 11.8061 (9) Å
*b* = 14.3419 (7) Å
*c* = 10.7891 (8) Å
β = 118.478 (3)°
*V* = 1605.78 (19) Å^3^
*Z* = 4
Cu *K*α radiation
μ = 0.50 mm^−1^
*T* = 173 K
0.50 × 0.20 × 0.05 mm

#### Data collection

Enraf–Nonius CAD-4 diffractometer
1607 measured reflections
1533 independent reflections
1165 reflections with *I* > 2σ(*I*)
*R*_int_ = 0.065
60 standard reflections every 60 min  intensity decay: 3%

#### Refinement

*R*[*F* ^2^ > 2σ(*F* ^2^)] = 0.057
*wR*(*F* ^2^) = 0.186
*S* = 1.07
1533 reflections
117 parameters
H-atom parameters constrained
Δρ_max_ = 0.31 e Å^−3^
Δρ_min_ = −0.23 e Å^−3^

### 

Data collection: *CAD-4 Software* (Enraf–Nonius, 1989 ▶); cell refinement: *CAD-4 Software*; data reduction: *CORINC* (Dräger & Gattow, 1971 ▶); program(s) used to solve structure: *SIR97* (Altomare *et al.*, 1999 ▶); program(s) used to refine structure: *SHELXL97* (Sheldrick, 2008 ▶); molecular graphics: *PLATON* (Spek, 2009 ▶); software used to prepare material for publication: *PLATON*.

## Supplementary Material

Crystal structure: contains datablock(s) I, global. DOI: 10.1107/S1600536811047672/bt5711sup1.cif

Structure factors: contains datablock(s) I. DOI: 10.1107/S1600536811047672/bt5711Isup2.hkl

Supplementary material file. DOI: 10.1107/S1600536811047672/bt5711Isup3.cml

Additional supplementary materials:  crystallographic information; 3D view; checkCIF report

## Figures and Tables

**Fig. 1.**
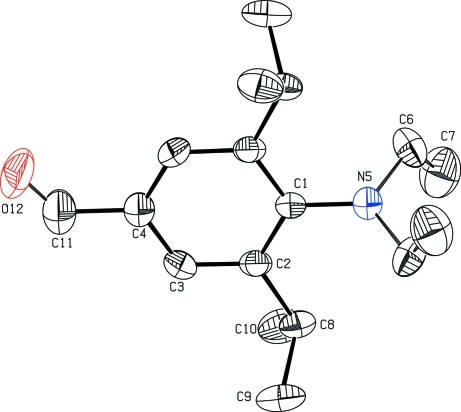
View of compound I. Displacement ellipsoids are drawn at the 50% probability level. Only major conformer is shown.

## supplementary crystallographic information

### Comment

The aldehyde forms needle-shaped crystals of the monoclinic space group *C*2/*c*.The compound crystallizes in parallel layers composed of geometrical dimers with *C*2 symmetry and *z* = 2. Two modes of disorder are present. A symmetry axis through C11—C4—C1—N4 results in a C2-symmetry of the molecule - but the carboxaldehyde group is disordered (50/50). The symmetry equivalent ethyl groups are also disordered but the s.o.f. is 60/40. The planes of the amino group and the benzene ring open an angle of 67.3 (3) ° whereas the carbonyl group and the aromatic system are essentially coplanar (torsion angle C3—C4—C11—O12: -179.0 (4) °). The sum of the bond angles around the nitrogen atom in both conformers is 359.9 (2) °, corresponding to a *sp*^2^ hybridization. With 1.414 (3) Å, the aniline C—N bond is much longer than the corresponding bond (1.366 (2) Å) in the sterically undisturbed dimethylaminobenzaldehyde (Gao & Zhu, 2008). This may be in part due to steric congestion, but also due to inhibited conjugation. Correspondingly, the carbonyl bond length of 1.114 (4) Å in the title compound is shorter than in the dimethylamino derivative (1.204 (2) Å, 1.212 (3) Å) and with 1.470 (4) Å the aryl-carbonyl bond C4—C11 is longer than in the reference compound (1.457 (3) Å, 1.454 (2) Å).

### Experimental

4-Bromo-*N*,*N*-diethyl-2,6-diisopropylaniline (0.76 g, 2.5 mmol) was dissolved in THF (15 ml) under nitrogen in a flame-dried Schlenk tube. The solution was cooled to195 K and *n*-BuLi (2.5 *M* in heptane, 1 ml) was added dropwise. After stirring for 1 h, dry DMF (0.2 ml, 2.5 mmol) was added carefully, stirring was continued for 15 min at 195 K, and the solution was allowed to reach room temperature. Aqueous NH_4_Cl (conc. 15 ml) was added and the mixture extracted with ethyl acetate (3 * 20 ml). The pooled organic solutions were dried (Na_2_SO_4_), concentrated *in vacuo* and the product was isolated *via* column chromatography (SiO_2_, petroleum ether / ethyl acetate = 15 / 1) *R*_f_ = 0.4 (petroleum ether / ethyl acetate = 9 / 1). The aldehyde was isolated as a yellowish oil that crystallized upon standing for several days. Yield: 0.23 g (35%)

### Refinement

Hydrogen atoms attached to carbons were placed at calculated positions with C—H = 0.95 Å (aromatic) or 0.98–0.99 Å (*sp*^3^ C-atom). All H atoms were refined in the riding-model approximation with isotropic displacement parameters  set at 1.2–1.5 times of the *U*_eq_ of the parent atom.

### Crystal data

**Table:**

C_17_H_27_NO	*F*(000) = 576
*M**_r_* = 261.40	*D*_x_ = 1.081 Mg m^−^^3^
Monoclinic, *C*2/*c*	Cu *K*α radiation, λ = 1.54178 Å
Hall symbol: -C 2yc	Cell parameters from 25 reflections
*a* = 11.8061 (9) Å	θ = 60–70°
*b* = 14.3419 (7) Å	µ = 0.50 mm^−^^1^
*c* = 10.7891 (8) Å	*T* = 173 K
β = 118.478 (3)°	Needle, colourless
*V* = 1605.78 (19) Å^3^	0.50 × 0.20 × 0.05 mm
*Z* = 4	

### Data collection

**Table:**

Enraf–Nonius CAD-4 diffractometer	*R*_int_ = 0.065
Radiation source: rotating anode	θ_max_ = 70.0°, θ_min_ = 5.3°
graphite	*h* = 0→14
ω/2θ scans	*k* = 0→17
1607 measured reflections	*l* = −13→11
1533 independent reflections	60 standard reflections every 60 min
1165 reflections with *I* > 2σ(*I*)	intensity decay: 3%

### Refinement

**Table:**

Refinement on *F*^2^	Secondary atom site location: difference Fourier map
Least-squares matrix: full	Hydrogen site location: inferred from neighbouring sites
*R*[*F*^2^ > 2σ(*F*^2^)] = 0.057	H-atom parameters constrained
*wR*(*F*^2^) = 0.186	*w* = 1/[σ^2^(*F*_o_^2^) + (0.1003*P*)^2^ + 0.8224*P*] where *P* = (*F*_o_^2^ + 2*F*_c_^2^)/3
*S* = 1.07	(Δ/σ)_max_ < 0.001
1533 reflections	Δρ_max_ = 0.31 e Å^−^^3^
117 parameters	Δρ_min_ = −0.23 e Å^−^^3^
0 restraints	Extinction correction: *SHELXL97* (Sheldrick, 2008), Fc^*^=kFc[1+0.001xFc^2^λ^3^/sin(2θ)]^-1/4^
Primary atom site location: structure-invariant direct methods	Extinction coefficient: 0.0061 (10)

### Special details

**Table:**

Experimental. ^1^H-NMR (400 MHz, CDCl_3_): 9.95 (s, 1 H, CHO), 6.61 (s, 2 H, 2-H, 6-H), 3.49 (sept, ^3^*J* = 6.9 Hz, 2 H, CH (i-Pr)), 3.11 (q, ^3^*J* = 7.1 Hz, 4 H, N-CH_2_), 1.22 (d, ^3^*J* = 6.9 Hz, 12 H, CH_3_ (iPr)), 1.04 (t, ^3^*J* = 7.1 Hz, 6 H, CH_3_ (Et)).^13^C-NMR (75 MHz, CDCl_3_): 192.5 (CHO), 152.3 (C-4), 150.9 (C-3, C-5), 134.3 (C-1), 126.0 (C-2, C-6), 48.9 (N-CH_2_), 29.2 (CH (iPr), 24.5 (CH_3_ (iPr)), 15.2 (CH_3_ (Et)).IR (ATR) ν = 2963, 2928, 2869, 2723, 1696, 1594, 1568, 1457, 1365, 1268, 1170, 1104, 1065, 941, 892, 783, 724 cm^-1^.HR-ESI-MS: found 262.2177, calc. 262.2171 for (M+H^+^).
Geometry. All e.s.d.'s (except the e.s.d. in the dihedral angle between two l.s. planes) are estimated using the full covariance matrix. The cell e.s.d.'s are taken into account individually in the estimation of e.s.d.'s in distances, angles and torsion angles; correlations between e.s.d.'s in cell parameters are only used when they are defined by crystal symmetry. An approximate (isotropic) treatment of cell e.s.d.'s is used for estimating e.s.d.'s involving l.s. planes.
Refinement. Refinement of *F*^2^ against ALL reflections. The weighted *R*-factor *wR* and goodness of fit *S* are based on *F*^2^, conventional *R*-factors *R* are based on *F*, with *F* set to zero for negative *F*^2^. The threshold expression of *F*^2^ > σ(*F*^2^) is used only for calculating *R*-factors(gt) *etc*. and is not relevant to the choice of reflections for refinement. *R*-factors based on *F*^2^ are statistically about twice as large as those based on *F*, and *R*- factors based on ALL data will be even larger.

### Fractional atomic coordinates and isotropic or equivalent isotropic displacement parameters (Å^2^)

**Table:**

	*x*	*y*	*z*	*U*_iso_*/*U*_eq_	Occ. (<1)
C1	0.5000	0.29898 (16)	0.2500	0.0301 (6)	
C2	0.49043 (16)	0.25001 (12)	0.13197 (17)	0.0329 (5)	
C3	0.49237 (18)	0.15335 (13)	0.13533 (18)	0.0407 (5)	
H3	0.4884	0.1198	0.0574	0.049*	
C4	0.5000	0.10460 (18)	0.2500	0.0445 (7)	
N5	0.5000	0.39757 (14)	0.2500	0.0394 (6)	
C6	0.3927 (4)	0.4517 (2)	0.2464 (5)	0.0477 (12)	0.595 (7)
H6A	0.4284	0.5063	0.3095	0.057*	0.595 (7)
H6B	0.3471	0.4125	0.2841	0.057*	0.595 (7)
C7	0.2975 (5)	0.4852 (3)	0.1028 (5)	0.0704 (16)	0.595 (7)
H7A	0.3405	0.5274	0.0668	0.106*	0.595 (7)
H7B	0.2275	0.5186	0.1082	0.106*	0.595 (7)
H7C	0.2620	0.4318	0.0391	0.106*	0.595 (7)
C6A	0.3813 (6)	0.4420 (4)	0.1519 (7)	0.0511 (19)	0.405 (7)
H6C	0.3242	0.3953	0.0827	0.061*	0.405 (7)
H6D	0.3999	0.4910	0.0997	0.061*	0.405 (7)
C7A	0.3127 (8)	0.4849 (6)	0.2250 (9)	0.084 (3)	0.405 (7)
H7D	0.2754	0.4355	0.2570	0.126*	0.405 (7)
H7E	0.2439	0.5260	0.1593	0.126*	0.405 (7)
H7F	0.3741	0.5212	0.3064	0.126*	0.405 (7)
C8	0.47720 (17)	0.29910 (13)	0.00066 (18)	0.0384 (5)	
H8	0.4765	0.3678	0.0159	0.046*	
C9	0.5913 (2)	0.27726 (19)	−0.0249 (2)	0.0577 (7)	
H9A	0.6715	0.2966	0.0573	0.087*	
H9B	0.5812	0.3111	−0.1085	0.087*	
H9C	0.5941	0.2101	−0.0399	0.087*	
C10	0.3504 (2)	0.27324 (18)	−0.1286 (2)	0.0535 (6)	
H10A	0.3516	0.2070	−0.1502	0.080*	
H10B	0.3397	0.3108	−0.2094	0.080*	
H10C	0.2786	0.2853	−0.1093	0.080*	
C11	0.5000	0.0021 (2)	0.2500	0.0730 (11)	
H11	0.4960	−0.0251	0.1677	0.088*	0.50
O12	0.5039 (6)	−0.0493 (2)	0.3294 (5)	0.1012 (16)	0.50

### Atomic displacement parameters (Å^2^)

**Table:**

	*U*^11^	*U*^22^	*U*^33^	*U*^12^	*U*^13^	*U*^23^
C1	0.0306 (11)	0.0327 (12)	0.0323 (12)	0.000	0.0193 (9)	0.000
C2	0.0347 (9)	0.0379 (10)	0.0326 (9)	0.0009 (7)	0.0213 (7)	0.0008 (6)
C3	0.0542 (11)	0.0377 (10)	0.0404 (10)	0.0018 (8)	0.0309 (9)	−0.0059 (7)
C4	0.0589 (17)	0.0334 (14)	0.0487 (15)	0.000	0.0317 (13)	0.000
N5	0.0500 (13)	0.0301 (11)	0.0438 (12)	0.000	0.0272 (10)	0.000
C6	0.051 (2)	0.0404 (19)	0.046 (2)	0.0115 (16)	0.0187 (17)	−0.0043 (15)
C7	0.062 (3)	0.062 (3)	0.073 (3)	0.008 (2)	0.020 (2)	0.017 (2)
C6A	0.063 (4)	0.037 (3)	0.054 (4)	0.012 (3)	0.028 (3)	0.008 (2)
C7A	0.068 (5)	0.082 (5)	0.092 (6)	0.020 (4)	0.031 (4)	−0.032 (4)
C8	0.0425 (10)	0.0476 (11)	0.0311 (9)	−0.0020 (8)	0.0225 (8)	0.0020 (7)
C9	0.0548 (13)	0.0874 (17)	0.0472 (12)	−0.0001 (11)	0.0374 (10)	0.0050 (11)
C10	0.0519 (12)	0.0748 (15)	0.0329 (10)	−0.0049 (10)	0.0194 (9)	0.0035 (9)
C11	0.112 (3)	0.0387 (17)	0.083 (3)	0.000	0.059 (2)	0.000
O12	0.195 (5)	0.0416 (19)	0.101 (3)	−0.003 (2)	0.098 (3)	0.0176 (19)

### Geometric parameters (Å, °)

**Table:**

C1—C2	1.411 (2)	C6A—C7A	1.505 (10)
C1—C2^i^	1.411 (2)	C6A—H6C	0.9900
C1—N5	1.414 (3)	C6A—H6D	0.9900
C2—C3	1.387 (3)	C7A—H7D	0.9800
C2—C8	1.522 (2)	C7A—H7E	0.9800
C3—C4	1.386 (2)	C7A—H7F	0.9800
C3—H3	0.9500	C8—C10	1.527 (3)
C4—C3^i^	1.386 (2)	C8—C9	1.530 (3)
C4—C11	1.470 (4)	C8—H8	1.0000
N5—C6A^i^	1.441 (6)	C9—H9A	0.9800
N5—C6A	1.441 (6)	C9—H9B	0.9800
N5—C6^i^	1.470 (4)	C9—H9C	0.9800
N5—C6	1.470 (4)	C10—H10A	0.9800
C6—C7	1.495 (6)	C10—H10B	0.9800
C6—H6A	0.9900	C10—H10C	0.9800
C6—H6B	0.9900	C11—O12^i^	1.114 (4)
C7—H7A	0.9800	C11—O12	1.114 (4)
C7—H7B	0.9800	C11—H11	0.9500
C7—H7C	0.9800		
			
C2—C1—C2^i^	120.3 (2)	C7A—C6A—H6D	109.2
C2—C1—N5	119.86 (11)	H6C—C6A—H6D	107.9
C2^i^—C1—N5	119.86 (11)	C6A—C7A—H7D	109.5
C3—C2—C1	118.74 (16)	C6A—C7A—H7E	109.5
C3—C2—C8	118.68 (15)	H7D—C7A—H7E	109.5
C1—C2—C8	122.58 (17)	C6A—C7A—H7F	109.5
C4—C3—C2	121.39 (17)	H7D—C7A—H7F	109.5
C4—C3—H3	119.3	H7E—C7A—H7F	109.5
C2—C3—H3	119.3	C2—C8—C10	111.02 (15)
C3^i^—C4—C3	119.4 (2)	C2—C8—C9	111.38 (15)
C3^i^—C4—C11	120.29 (12)	C10—C8—C9	110.43 (16)
C3—C4—C11	120.29 (12)	C2—C8—H8	108.0
C1—N5—C6A^i^	116.2 (2)	C10—C8—H8	108.0
C1—N5—C6A	116.2 (2)	C9—C8—H8	108.0
C6A^i^—N5—C6A	127.5 (5)	C8—C9—H9A	109.5
C1—N5—C6^i^	121.86 (16)	C8—C9—H9B	109.5
C6A—N5—C6^i^	108.1 (3)	H9A—C9—H9B	109.5
C1—N5—C6	121.86 (16)	C8—C9—H9C	109.5
C6A^i^—N5—C6	108.1 (3)	H9A—C9—H9C	109.5
C6^i^—N5—C6	116.3 (3)	H9B—C9—H9C	109.5
N5—C6—C7	114.2 (4)	C8—C10—H10A	109.5
N5—C6—H6A	108.7	C8—C10—H10B	109.5
C7—C6—H6A	108.7	H10A—C10—H10B	109.5
N5—C6—H6B	108.7	C8—C10—H10C	109.5
C7—C6—H6B	108.7	H10A—C10—H10C	109.5
H6A—C6—H6B	107.6	H10B—C10—H10C	109.5
N5—C6A—C7A	112.0 (6)	O12^i^—C11—C4	131.4 (3)
N5—C6A—H6C	109.2	O12—C11—C4	131.4 (3)
C7A—C6A—H6C	109.2	O12—C11—H11	114.3
N5—C6A—H6D	109.2	C4—C11—H11	114.3
			
C2^i^—C1—C2—C3	−0.85 (12)	C1—N5—C6—C7	−97.8 (3)
N5—C1—C2—C3	179.15 (12)	C6A^i^—N5—C6—C7	123.5 (4)
C2^i^—C1—C2—C8	178.76 (17)	C6A—N5—C6—C7	−4.4 (4)
N5—C1—C2—C8	−1.24 (17)	C6^i^—N5—C6—C7	82.2 (3)
C1—C2—C3—C4	1.7 (2)	C1—N5—C6A—C7A	107.0 (5)
C8—C2—C3—C4	−177.89 (13)	C6A^i^—N5—C6A—C7A	−73.0 (5)
C2—C3—C4—C3^i^	−0.89 (12)	C6^i^—N5—C6A—C7A	−111.7 (5)
C2—C3—C4—C11	179.11 (12)	C6—N5—C6A—C7A	−2.0 (4)
C2—C1—N5—C6A^i^	−112.2 (3)	C3—C2—C8—C10	61.2 (2)
C2^i^—C1—N5—C6A^i^	67.8 (3)	C1—C2—C8—C10	−118.38 (18)
C2—C1—N5—C6A	67.8 (3)	C3—C2—C8—C9	−62.3 (2)
C2^i^—C1—N5—C6A	−112.2 (3)	C1—C2—C8—C9	118.10 (18)
C2—C1—N5—C6^i^	−67.7 (2)	C3^i^—C4—C11—O12^i^	−179.0 (4)
C2^i^—C1—N5—C6^i^	112.3 (2)	C3—C4—C11—O12^i^	1.0 (4)
C2—C1—N5—C6	112.3 (2)	C3^i^—C4—C11—O12	1.0 (4)
C2^i^—C1—N5—C6	−67.7 (2)	C3—C4—C11—O12	−179.0 (4)

Symmetry codes: (i) −*x*+1, *y*, −*z*+1/2.
